# Anticancer Potential of Betulonic Acid Derivatives

**DOI:** 10.3390/ijms22073676

**Published:** 2021-04-01

**Authors:** Adelina Lombrea, Alexandra Denisa Scurtu, Stefana Avram, Ioana Zinuca Pavel, Māris Turks, Jevgeņija Lugiņina, Uldis Peipiņš, Cristina Adriana Dehelean, Codruta Soica, Corina Danciu

**Affiliations:** 1 Department of Pharmacognosy, “Victor Babes” University of Medicine and Pharmacy, Eftimie Murgu Square, No. 2, 300041 Timisoara, Romania; lombrea.adelina@yahoo.com (A.L.); stefana.avram@umft.ro (S.A.); ioanaz.pavel@umft.ro (I.Z.P.); corina.danciu@umft.ro (C.D.); 2 Research Centre for Pharmaco-Toxicological Evaluation, “Victor Babes” University of Medicine and Pharmacy, Eftimie Murgu Square, No. 2, 300041 Timisoara, Romania; cadehelean@umft.ro (C.A.D.); codrutasoica@umft.ro (C.S.); 3 Department of Toxicology, “Victor Babes” University of Medicine and Pharmacy, Eftimie Murgu Square, No. 2, 300041 Timisoara, Romania; 4 Institute of Technology of Organic Chemistry, Faculty of Materials Science and Applied Chemistry, Riga Technical University, P. Valdena Str. 3, LV-1048 Riga, Latvia; maris.turks@rtu.lv (M.T.); jevgenija.luginina@rtu.lv (J.L.); 5 Nature Science Technologies Ltd., Saules Str. 19, LV-3601 Ventspils, Latvia; sales@clickbetulin.com; 6 Department of Pharmaceutical Chemistry, “Victor Babes” University of Medicine and Pharmacy, Eftimie Murgu Square, No. 2, 300041 Timisoara, Romania

**Keywords:** pentacyclic triterpenes, betulonic acid derivatives, natural compounds, proliferation, apoptosis, in vitro, in vivo, cancer

## Abstract

Clinical trials have evidenced that several natural compounds, belonging to the phytochemical classes of alkaloids, terpenes, phenols and flavonoids, are effective for the management of various types of cancer. Latest research has proven that natural products and their semisynthetic variants may serve as a starting point for new drug candidates with a diversity of biological and pharmacological activities, designed to improve bioavailability, overcome cellular resistance, and enhance therapeutic efficacy. This review was designed to bring an update regarding the anticancer potential of betulonic acid and its semisynthetic derivatives. Chemical derivative structures of betulonic acid including amide, thiol, and piperidine groups, exert an amplification of the in vitro anticancer potential of betulonic acid. With the need for more mechanistic and in vivo data, some derivatives of betulonic acids may represent promising anticancer agents.

## 1. Introduction. Natural Compounds in the Management of Different Types of Cancer

The extensive research over the past decades has identified an increased number of natural compounds from various medicinal plants that elicit chemopreventive potential. These phytochemicals represent an important potential source of anticancer molecules as such or after various physico-chemical modulations, which can be successfully used in different therapeutic protocols [1,2]. The analysis of natural compounds has led to the design of novel therapeutics owing to their vast structural diversity, supported by the fact that plants are accessible resources. The main issues are the isolation and purification of bioactive derivatives [3].

Cancer is one of the largest causes of mortality in the world and due to its prevalence, the discovery of novel anticancer drugs has great importance. Plants, microorganisms, and marine organisms are considered valuable sources for the discovery of anticancer drugs [4,5].

Throughout history, natural products and their derivatives have proved to be useful for the development of chemotherapeutics, presenting a great structural diversity as well as molecular and pharmacological properties that favor such development [6].

Plants have a long history of use in the treatment of cancer. Numerous classes of natural compounds such as alkaloids, terpenes, phenols, and flavonoids have been shown to be effective in clinical trials [7]. Vinca alkaloids: vincristine, vinblastine, vindesine, and vinorelbine isolated from *Vinca rosea* L. (*Apocynaceae*), were the first agents to advance into clinical use. At low doses, they interrupt microtubular activity, whereas, at higher doses, they cause cell cycle arrest and apoptosis [8,9]. Vinca alkaloids have been licensed by the Food and Drug Administration (FDA) as a pharmaceutical treatment against different tumors (leukemia, Hodgkin’s lymphoma, lung cancer, breast cancer) [10]. Five vinca alkaloids are actually in clinical usage: vinblastine and vincristine were approved by the FDA in 1961 and 1963, respectively; the semi-synthetic derivatives vindesine and vinorelbine were approved by the FDA in 1994, and vinflunine, a synthetic derivative, was approved by EMA (European Medicines Agency) in 2012 for treatment of metastatic and advanced urothelial cancer [11,12]. Moreover, in 2019, World Health Organization included vincristine, vinblastine, and vinorelbine on the list of essential medicines for treating Hodgkin lymphoma, Kaposi sarcoma, testicular germ cell tumor, ovarian germ cell tumor, follicular lymphoma, retinoblastoma, rhabdomyosarcoma, Ewing sarcoma, acute lymphoblastic leukemia, non-small cell lung cancer, and metastatic breast cancer [13].

The alkaloid piperine, isolated from the fruits of *Piper nigrum* L. in combination with curcumin, is involved in an ongoing phase I clinical trial for the treatment of bladder cancer [14]. Also, camptothecin, a quinoline alkaloid isolated especially from the stem and bark of *Camptotheca acuminata* Decne, has completed phase III clinical trials for the treatment of pancreatic, ovarian, and colorectal cancers [15]. Currently, irinotecan and topotecan are the two FDA-licensed semi-synthetic camptothecin derivatives and are clinically active and less harmful than the parent compound. Irinotecan is used for advanced cancers of the large intestine and rectum. Topotecan is approved for the treatment of recurrent ovarian cancer, small cell lung cancer, and cancer of the cervix [16].

Terpenes are another class of natural compounds introduced in clinical trials. Ursolic acid, a pentacyclic triterpenoid widely found in different species of plants, has completed phase II clinical trials for the treatment of solid tumors [17]. Paclitaxel, a diterpenoid, isolated from the bark of *Taxus brevifolia* Nutt and its derivatives such as nanopaclitaxel and docetaxel have completed phases I, II, III, and IV of clinical trials for the treatment of breast, ovarian, and endometrial cancers [18,19]. The FDA approved paclitaxel for the treatment of refractory ovarian cancer in 1992, refractory breast cancer in 1994, Kaposi’s sarcoma in 1997, and non-small cell lung cancer in 1998. Docetaxel, a semi-synthetic derivative of paclitaxel, showed greater effectiveness than paclitaxel in certain instances. The FDA authorized docetaxel for the treatment of advanced breast cancer in 1996, non-small pulmonary cancer in 1999, metastatic hormone-refractory prostate cancer in 2004, and head and neck cancer in 2006 [20]. Several of the paclitaxel analogs, such as larotaxel, milataxel, ortataxel, and tesetaxel, are currently under investigation in clinical trials [16].

Lycopene, a carotenoid type terpene, majorly found in red fruits and vegetables, has completed phase II clinical trial for the treatment of hormone-resistant prostate cancer in combination with docetaxel [21,22]. Currently, lycopene is in phase II regarding its effectiveness to reduce skin toxicity in patients with colorectal carcinoma treated with panitumumab [23].

Among phenolic compounds, one of the largest classes of plant secondary metabolites, curcumin, isolated from *Curcuma* plant species, has completed phase I clinical trials for treating metastatic breast cancer in co-therapy with docetaxel [24]. Moreover, curcumin along with paclitaxel has demonstrated efficacy and safety in phase II clinical trials in patients with advanced, metastatic breast cancer [25]. Currently, this phenolic compound is involved in phase III clinical trials regarding its potential to reduce cancer progression in patients with low-risk prostate cancer under active surveillance [26]. Chlorogenic acid has passed phase I clinical study for treating malignant gliomas. The findings revealed that monotherapy of chlorogenic acid in patients with advanced malignancies was effective and well-tolerated for long-term use. Interestingly, for subjects treated with chlorogenic acid, the median overall survival with grade IV gliomas was 21.4 months, while the globally recognized median overall survival was just 3–4.6 months for subjects at this point [27].

Flavonoids are secondary metabolites commonly found in plants [28]. Flavonoids are usually found in citrus fruits, berries, legumes, green tea, as well as in red wine [29]. Genistein along with cholecalciferol has completed phase II clinical trials treating patients with early-stage prostate cancer [30]. Quercetin is classified as a flavonol and is currently involved in an ongoing phase I trial, which is evaluating its impact on green tea polyphenol absorption in the prostate tissue from patients with prostate cancer [31,32]. Moreover, quercetin is also involved in an ongoing phase II clinical trial regarding its chemopreventive activity in the development of squamous cell carcinoma in patients with Fanconi anemia [33].

Although there are many studies on betulin and betulinic acid [34,35], this review focuses on betulonic acid and, more specifically, on its derivatives, which have also shown important therapeutic effects, thus suggesting their further thorough research.

## 2. An Overview of the Biologic Activity of Pentacyclic Triterpenes

**Figure 1:**
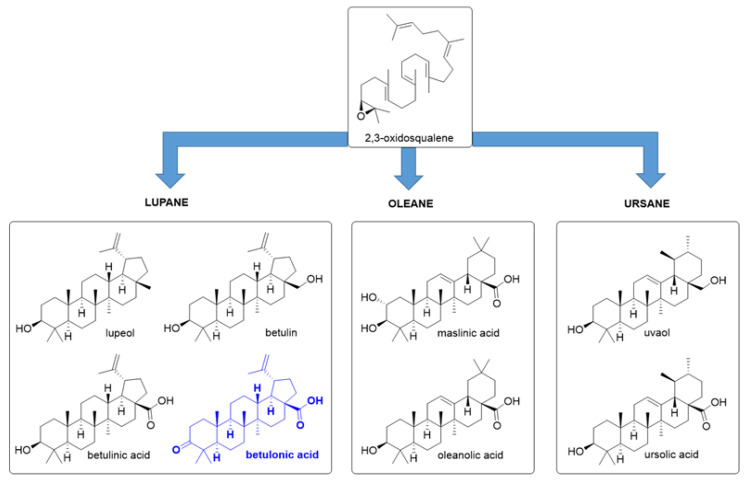
Biologically active pentacyclic triterpenoids in the management of different types of cancer.

To date, more than 20,000 triterpenes have been isolated [36]. Triterpenoids are originally synthesized by plants as metabolites and are abundantly present in most medicinal plants in the form of free acids or aglycones. Triterpenes are a diverse group of natural compounds, classified into two main groups: tetracyclic and pentacyclic triterpenes. The class of tetracyclic triterpenes includes compounds such as: euphol, oleandrin, and cucurbitacin. The class of pentacyclic triterpenoids (PT) is richer in structures representative for their biological activity, lupane, oleane, and ursane scaffolds being the main groups of this class. Important phytocompounds due to their biological activity and belonging to the category of pentacyclic triterpenoids are: lupeol, betulin, betulinic acid, betulonic acid (lupane structure), oleanolic acid, maslinic acid (oleanane structure), ursolic acid, and uvaol (ursane structure) (Figure 1), nevertheless other such categories including hopane, serratane, friedelane, and taraxane can also be referred [37,38,39].

Secondary plant metabolites such as PT are generated through the cyclization of squalene [40]. Lupeol, oleanane, and ursane structures were found in a variety of plant parts such as bark, cork, leaf, or fruit cuticular wax [41]. Moreover, pentacyclic triterpenes have been detected in edibles such as mango, apple peel, strawberries, pear peel, green pepper, guava, mulberry, or olives, but also in herbal plants such as rosemary, oregano, basil, and lavender. The individual natural human consumption of triterpenes is reported to be approximately 250 mg per day in the Western world and 400 mg per day in Mediterranean countries [42]. However, small quantities, estimated to be under 0.1% of the dry weight of the plant organ, are found in plants. Even so, few plants are considered to have elevated amounts of pentacyclic triterpenes, above 1% of the dry weight of the plant. The highest pentacyclic terpene content has been noticed in the outer bark of the white birch tree (up to 34% [*w*/*w*] in betulin) [40]. Furthermore, the leaves from *Rosmarinus officinalis* L., *Olea europaea* L., *Coffea arabica* L., the sapling from *Viscum album* L., the bark from *Platanus* L., and the fruit peels from *Malus domestica* Mill. produce more than 1% (*w*/*w*) pentacyclic triterpenes. Due to this property, these plants are useful for obtaining triterpene dry extracts which contain 50–90% (*w*/*w*) triterpenes [43].

Natural PT have a broad range of biological activities. Intense pharmacological researches have been conducted on natural PT in order to exploit their medicinal value and mechanism of action. In general, the bioactivities of these phytocompounds have been shown to include antitumor [40,44,45], antiviral [46], antidiabetic [47], anti-inflammatory [48], antimicrobial [49], antiparasitic [39], cardio-protective [50], hepato-protective [51], wound healing properties [52], and others. For example, oleanolic acid, glycyrrhizin, glycyrrhetinic acid, ursolic acid, betulin, betulinic acid, and lupeol increase the absorption of glucose, enhance insulin secretion, boost glucose uptake in peripheral organs, and contribute to the treatment of diabetes and its vascular complications [53]. Zhang et al. tested the inhibitory activity of oleanane-, ursane- and lupane-type triterpenes against *α*-amylase and yeast *α*-glucosidase. Results have shown that, in comparison to the IC_50_ value of acarbose (5.3 µM), ursolic acid exerted the highest inhibition of *α*-amylase, with an IC_50_ value of 22.6 μM, followed by corosolic acid and oleanolic acid with IC_50_ values of 31.2 µM and 94.1 µM, respectively. Regarding the *α*-glucosidase inhibition, ursolic acid, betulinic acid, and corosolic acid displayed a stronger inhibitory activity than acarbose (IC_50_ = 2479 µM) with IC_50_ levels of 12.1 µM, 14.9 µM, and 17.2 µM, respectively. It appears that the variety of structural skeletons of pentacyclic triterpenes had a substantial effect on the inhibition of *α*-amylase activity. The fact that ursolic acid displayed stronger *α*-amylase inhibition than oleanolic acid may be attributted to the shift of the C-29 methyl group from C-20 to C-19. The study found that betulinic acid inhibited *α*-glucosidase with comparable effectiveness to ursolic acid, indicating that the disparity between ursane and lupane had no major effect on the enzyme inhibition [54]. Numerous experiments have shown that different PT have antidiabetic properties in healthy or diabetic animal models. Phanoside (a dammarane-type structure) at a dosage of up to 500 μM exerted significant effects on insulin secretion in rat pancreatic cells. In INS-1 832/13 pancreatic *β*-cells, oleanolic acid (50 μM) improved insulin secretion. Moreover, in isolated rat islets, oleanolic acid (30 μM) also improved insulin secretion, which was stimulated by high glucose levels [55,56]. The plasma insulin levels of Wistar rats were improved by ginsenoside Rh2 (1.0 mg/kg) and oleanolic acid (5, 10 and 20 mg/kg) by freeing acetylcholine (ACh) from nerve terminals and then activating M3 muscarinic receptors in pancreatic cells [57,58].

Furthermore, PT showed anti-inflammatory, antioxidant, anti-adiposity, and cardioprotective properties in high-carbohydrate high-fat diet-induced metabolic syndrome [59,60]. In addition, the weight-reducing effects of PT are due to the downregulation of pro-inflammatory cytokines, insulin resistance, oxidative stress, and total fatty acids, all of these contributing to a more efficient glycemic regulation [61]. In the study performed by Tang et al., they have demonstrated that betulin at 3 µg/mL incubated for 6 h suppressed the maturation of sterol regulatory element-binding proteins (SREBP) and reduced cholesterol and fatty acid biosynthesis in rat hepatocytes CRL-1601. At a dosage of 30 mg/kg/day for 6 weeks, betulin has lowered serum and tissue lipid levels, helped improve glucose tolerance, and tended to increase insulin sensitivity in C57BL/6J mice fed with Western-style diet. In addition, the application of betulin to the LDLR-knockout mice model of atherosclerosis disease has revealed its ability to decrease the size of the atherosclerotic plaques [62]. As well as betulin, betulinic acid (50 mg/kg for 15 weeks) lowered body weight, blood glucose, abdominal fat, plasma triglycerides, and total cholesterol levels in Swiss mice fed with a high-fat diet. Interestingly, betulinic acid treatment further decreased the circulating level of the orexigenic hormone ghrelin and increased the anorexigenic hormone leptin. The results showed that betulinic acid could represent a candidate to cure obesity through changes in fat and carbohydrate metabolisms [63].

Several in vitro and in vivo studies have shown that natural triterpenes such as lupeol, betulin, ursolic acid, and oleanolic acid are successful in reducing hepatotoxicity caused by carbon tetrachloride, acetaminophen, ethanol, and cadmium [64,65,66]. In vitro, betulinic acid has been shown to be a powerful antioxidant agent, which can suppress ethanol-induced activation of hepatic stellate cells mostly by the repression of reactive oxygen species (ROS) and tumor necrosis factor-*α* (TNF-*α*) [67]. In the in vivo experiments performed by Yi et al., the authors have highlighted the hepatoprotective effects of betulinic acid, when given orally (0.25, 0.5, and 1.0 mg/kg daily for 14 days) to Kunming mice with induced alcoholic liver disease. Betulinic acid increased hepatic glutathione, superoxide dismutase, glutathione peroxidase, and catalase levels, as well as the levels of malondialdehyde, thereby decreasing the liver’s microvesicular steatosis. In addition, the results of the study indicated that betulinic acid-treated mice showed reduced serum levels of triglycerides and total cholesterol, which was attributed to enhanced *β*-oxidation in the liver and energy consumption. Betulinic acid also reduced the accumulation of visceral adipose tissue in alcohol-treated mice. In particular, this may help prevent secondary complications emerging from elevated levels of hepatic lipids [68].

PT derived from natural products exhibit a wide variety of medicinal properties such as anti-oxidant [69], anti-tumor [70], anti-microbial [71], and anti-inflammatory [72]. It is assumed that the pathways involved in these bioactivities are caused by the regulation of the immune system. The immunomodulatory efficacy of botanical pentacyclic triterpenes extracted from a broad variety of medicinal plant species, based on different in vitro and in vivo experimental models, has been illustrated in several reports [73,74]. In the experiment conducted by Saaby et al., the immunomodulatory effects of various PT obtained from *Rosa canina* L. have been evaluated in vitro, on lipopolysaccharide (LPS)-activated Mono Mac 6 cells (an in vitro model for human macrophages). The findings showed that oleanolic, betulinic, and ursolic acid mixtures (30:49:21 *w*/*w*) inhibit the release of LPS-induced IL-6 in a stronger manner than individual compounds, with an IC_50_ value of 21 μmol/L [75]. In the study conducted by Ayatollahi et al., the authors investigated the impact of three phytocompounds (betulinic acid, oleanolic acid and ursolic acid), extracted from *Euphorbia microsciadia* Boiss, on T-cell proliferation. The T-lymphocytes were isolated from healthy volunteers and analysed using a liquid scintillation counter. Studies have shown that oleanolic acid induces T cell proliferation even at the concentration of 0.5 μg/mL. These findings indicated that oleanolic acid has an important immunostimulating action at very low concentration. Betulinic acid and ursolic acid, on the other hand, were able to suppress the proliferation of T cells with IC_50_ values above 50 μg/mL and 3 μg/mL, respectively [76]. Marquez-Martin et al. observed that, at concentrations of 10, 25, 50, and 100 μmol/L, some PT extracted from ‘orujo’ olive oil such as oleanolic acid and erythrodiol dose-dependently decreased the secretion of IL-1*β* and IL-6 isolated from peripheral mononuclear blood cells (PBMCs) of healthy volunteers. Erythrodiol exhibited the most potent inhibitory effect (*p* < 0.05) on the development of IL-1*β* and IL-6 PBMCs at all doses. At the maximum tested doses (50–100 μM), oleanolic acid substantially (*p* < 0.05) down-regulated IL-1*β* and IL-6 release [77].

The molecular mechanisms behind the different biological activities of PT, ranging from inhibition of acute and chronic inflammation, inhibition of tumor cell proliferation, activation of apoptosis, and suppression of angiogenesis and metastasis, have been identified in various research studies [40,78]. PT modulate a wide range of molecular targets (cytokines; chemokines; reactive oxygen intermediates; oncogenes; inflammatory enzymes; anti-apoptotic proteins; and transcription factors such as NF-jB, STAT3, AP-1, and CREB), which mediate tumor cell proliferation, transformation, invasion, angiogenesis, metastasis, chemoresistance, and radioresistance [78]. Previous studies have demonstrated that PT, in particular betulin, betulinic acid, lupeol, and ursolic acid, have caused apoptosis in various forms of cancer cells by stimulation of the mitochondrial pathway (intrinsic pathway) rather than the death receptor pathway (extrinsic way) [79,80].

Betulinic acid is highly promising due to its low toxicity in healthy cells and strong anti-proliferative effects against human melanoma cells (IC_50_ = 1.5–1.6 μg/mL) [81,82]; neuroblastoma (IC_50_ = 14–17 µg/mL); medulloblastoma (IC_50_ = 3–13.5 µg/mL); Ewing’s sarcoma; leukemia; brain-tumors; glioblastoma (IC_50_ = 2–17 μg/mL);colon carcinoma; ovarian cancer (IC_50_ = 1.8–4.5 μg/mL); lung cancer (IC_50_ = 1.5–4.2 μg/mL); breast, prostate, head and neck squamous cell carcinoma and hepatocellular; and renal and cervical cancers (IC_50_ = 1.8 μg/mL) [34]. In addition, cell death via the endoplasmic reticulum pathway and ROS-mediated mitochondrial pathway was observed in cervix adenocarcinoma cells (HeLa) treated with betulinic acid [83,84,85]. In nude mice xenografts bearing estrogen receptor-negative MDA-MB-231 cells, betulinic acid has prevented tumor growth by decreasing tumor size and reducing the expression of mRNA of Sp transcription factors, which control the expression of genes responsible for cancer growth (SP1, SP3, SP4, VEGFR, and miRNA-27) [86]. In another research study, betulinic acid along with vincristine was able to inhibit lung metastases in an experimental model including B16F10 murine melanoma cells injected in C57BL/6 mice strain [87].

Betulonic acid extracted from *Toona sinensis* (A Juss.) M.Roem and *Belamcanda chinensis* L. at 20 μmol/L has shown substantial antitumor activity against various types of cancer cells: human gastric cancer cell line MGC-803 (Inhibitory rate = 56% for *Toona sinensis* (A Juss.) and 68% for *Belamcanda chinensis* L.), prostatic cancer cell line PC3 (Inhibitory rate = 63% for *Toona sinensis* Juss and 52% for *Belamcanda chinensis* L.), and breast cancer cell line MCF-7 (Inhibitory rate = 51% for *Toona sinensis* Juss and 56% for *Belamcanda chinensis* L.) [83,88]. This antitumor activity includes the potential of betulonic acid to cause apoptosis through the mitochondrial signalling cascade, which involves the expression of caspases 3 and 9 and proteins p53 and Bax [83].

## 3. Betulonic Acid and Its Derivatives. An Overview of In Vitro and In Vivo Active Compounds

**Figure 2:**
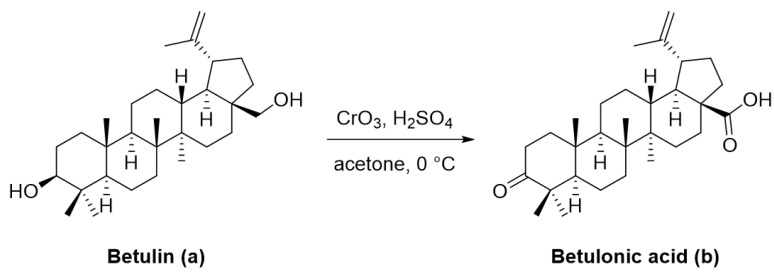
Semi-synthesis of betulonic acid.

Betulin (lup-20(29)-ene-3*β*,28-diol) and betulonic acid (lup-20(29)-en-3-oxo-28-oic) (Figure 2) are the most important pentacyclic lupane-structure triterpenoids of natural origin. These compounds are found in a broad range of medicinal plants, but they are often extracted from birch bark through sublimation or extraction with organic solvents including ethanol, acetone, and chloroform [37,89]. Betulonic acid has a keto group at C-3 and a carboxyl group at C-28, with a terminal double bond at C-29; the only structural difference compared to betulinic acid lies at the C-3 position where betulonic acid bears a ketone instead of a *β*-configured hydroxyl [83].

*Betulaceae* family is the richest source of betulin, with the most important representatives being *Betula alba* L., *Betula pendula* Roth, *Betula pubescens* Ehrh. and *Betula platyphylla* Suk. [90]. Betulin has also been found in various species of the genera *Syzygium* (*Myrtaceae*) [91], *Doliocarpus (Dilleniaceae)* [92], *Paeonia* (*Paeoniaceae*) [93], and *Ziziphus* (*Rhamnaceae*) [94], being extracted from bark, leaves, roots, twigs, and fruits. Betulonic acid was traditionally isolated from the fruit of *Liquidambar formosana* Hance trees with an abundance of 13.4% [95].

The content of betulonic acid in plants is generally low, so it is frequently obtained by a semisynthetic process, which consists in the oxidation of betulin (Figure 2), the main constituent (22–30%) found in the outer part of birch bark [80].

Betulonic acid and its derivatives have been found to possess several medicinal properties, such as anti-viral [96,97,98], antimicrobial [99,100], anti-Human cytomegalovirus (HCMV) [3], anti-inflammatory [101,102,103] antioxidant [104], hepatoprotective [103,105], immunostimulant [106], and anticancer effects [107,108]. Modern research has proved that natural products and their semisynthetic variants may serve as the starting point of new drug candidates with a diversity of biological and pharmacological activities. From 1981–2014, natural products accounted for 25% of all newly licensed medicines [109,110]. Betulonic acid is an example of a natural compound that was found to be a promising candidate as an antitumor agent since it can inhibit the growth of different types of tumor cell lines [83]. Betulonic acid is soluble in organic solvents, but its solubility in water is weak. Chemical modifications are used to obtain derivatives with higher bioavailability and special selectivity to cellular targets [111]. Several derivatives were synthesized and examined against different cancer cell lines for antitumor activity (Table 1).

The group conducted by Shintyapina et al. examined the inhibitory action of selected amides of betulonic acid on the growth and potential apoptosis induction of MT-4, MOLT-4, CEM (lymphoblastic leukemia), and Hep G2 (liver cancer) tumor cell lines. Betulonic acid amides (compounds I (A–D)) inhibited cell growth at low concentrations (50% inhibition [ID_50_] = 4.2–32.0 µg/mL), as shown in Table 2. The length of the methylene moiety (CH_2_)_n_ in the amide residue did not affect the inhibitory activity, however, the introduction of the second amide residue increased the inhibition of the tumor cell growth (I (D)). The apoptosis-inducing activity of betulonic acid amides was higher than that of betulonic acid for MT-4 and MOLT-4 cell lines. Thus, the introduction of amide function at the position C28 of PT led to the formation of effective inducers of apoptosis [112].

Yang et al. have pointed towards the in vitro antiproliferative ability of betulonic acid derivatives against A375 (malignant melanoma), PC3 (prostate cancer), MGC-803 (gastric cancer), MCF-7, and Bcap-37 (breast adenocarcinoma) cell lines. The compounds substituted with a bromoalkyl moiety (II (A), II (B)) demonstrated weak antitumor activity compared with the parent compound betulonic acid. Results have shown that (4-(piperidin-1-yl) butyl) 3-oxo-20(29)-lupen-28-oate (III) was one of the most active compounds, presenting IC_50_ values of 3.6–7.8 µM on the five screened cancer cell lines, whereas compounds substituted with fluorophenyl group (IV) exhibited high and moderate antiproliferative activities, as shown in Table 2. (4-(piperidin-1-yl)butyl) 3-oxo-20(29)-lupen-28-oate has also been shown to induce apoptosis of MGC-803 cells via the mitochondrial intrinsic pathway, which include suppression of the expressions p53, Bax, caspase 9, and caspase 3 [113].

In a similar approach, Ledeţi et al. described the cytotoxic effects (MTT assay) of four amino derivatives of betulonic acid against certain tumor cell lines, including HeLa (cervix adenocarcinoma), A431 (skin carcinoma), A2780 (ovarian carcinoma) and MCF-7 (breast adenocarcinoma), as shown in Table 2. Guanylhydrazone (V) had lower cytotoxic activity on all cell lines relative to betulin at both tested concentrations (10, 30 mM). The oxime (VI) was found to have strong cytotoxic effects on the A2780 cell line when used at elevated doses, whereas the cytotoxic effect on the A431 cells was almost negligible when the lower concentration was used. The strongest activity manifested at the lowest concentration of butyl imine (VII) occurred on HeLa cell lines, as opposed to the higher concentration that was relevant for A2780 cell line. Moreover, the thiosemicarbazone (VIII) demonstrated significant cytotoxic effects against HeLa, A2780, and MCF-7 cell lines included in this study, having a comparable inhibitory activity to betulin. Regarding the inhibitory activity against A431 cell line, there was no evidence of it [114].

The inhibitory effects of C(2)-C(3)-fused triazine derivatives of betulonic acid on the proliferation of murine leukemia cells (L1210), human cervix carcinoma cells (HeLa), and human T-lymphoblast cells (CEM) were evaluated by Dinh Ngoc et al. The 1,2,4-triazine derivative of betulonic acid (IX) did not elicit a significant cytostatic activity, while S-alkylated triazine derivatives (X, XI) strongly inhibited tumor cell proliferation, especially in case of human T-lymphoblast cells [3].

Kommera et al. demonstrated the in vitro cytotoxic activity of the betulonic acid derivative, 2-amino-3-hydroxy-2-(hydroxymethyl) propyl betulonate (XII), on eight different tumor cell lines: 518A2 (melanoma), A253 (head and neck tumor), A431 (cervical), A2780 (ovarian), A549 (lung), HT-29 (colon), MCF-7 (breast), and SW1736 (anaplastic thyroid tumor) using the sulforhodamine B colorimetric assay. As shown in Table 2, IC_50_ values were found to be in the range of 9.44–10.83 µM. A comparison regarding the cytotoxicity of betulonic acid and compound XII showed a loss of specificity towards some cancer cell lines presented by the parent compound. It has been observed that the derivate also induced apoptosis on HT-29 cell line, which has been confirmed by annexin V staining experiments and DNA fragmentation assay [115].

Following the study conducted by Saxena et al., it has been reported that N-Boc-lysinated-betulonic acid (XIII) elicits positive in vitro effects against prostate cancer cell lines (LNCaP, DU-145, PC-3). The growth of the LNCaP cells was inhibited by lysinated-betulonic acid at 10 μM following 72 h of incubation to 40%, whereas at a concentration of 100 μM, the inhibitory activity increased to 80% (for the compound dissolved in DMSO) and by 96% (for compound dissolved in phosphate-buffered saline [PBS]). Using the MTT assay and various incubation times, the research group showed a significant inhibitory effect on DU-145 and PC-3 cancer cells treated with 100 μM of lysinated-betulonic, as shown in Table 2 [116].

Antimonova et al. have synthesized and evaluated the in vitro cytotoxicity of betulonic acid bearing an 1,3,4-oxadiazole moiety in the C-17 position. The technique used for the preparation of the compounds involved the reaction of acid chlorides (betulonic acid chloride and 3-*β*-O-acetyl-betulinic acid chloride) with acid hydrazides accompanied by the cyclization of the resulting acylhydrazides (XIV (A–F)) and thus the synthesis of 1,3,4-oxadiazole derivatives (XV (A–F)). The cytotoxicity against human cancer cell line (MT-4), human T-cell leukemia (CEM-13), and human monocytes (U-937) was determined by MTT assay, and the results were expressed as ID_50_. Betulonic acid hydrazide (XIV (D)) with a 6-(trifluoromethyl)-2-fluoro-3-chlorophenyl substituent was the most powerful inhibitor of MT-4 (ID_50_ = 9 µM) and U-937 (ID_50_ = 12 µM) cells. This compound was slightly more active against U-937 human tumour cells than betulonic acid (ID_50_ =19 µM). Regarding the inhibitory activity against the CEM-13 cell line, mild cytotoxicity (ID_50_ = 32 µM) was noticed compared to betulonic acid (ID_50_ = 12 µM). The derivative with the substituent 4-Bromophenyl (XIV (E)) was the second hydrazide that has shown great inhibitory activity against MT-4 cell line (ID_50_ = 14 µM) and moderate cytotoxicity against U-937 and CEM-13 cell lines, with ID_50_ = 23 µM and ID_50_ = 23 µM, respectively. On the other hand, hydrazides with phenolic (XIV (B)) and pyridyl (XIV (F)) presented moderate cytotoxicity against MT-4 cell line and U-937 cell line; ID_50_ values were within 19–32 µM. Concerning the cytotoxicity against the CEM-13 cell line, ID_50_ values were high (ID_50_ = 57 µM for derivative (XIV (F)) and 72 µM for derivative (XIV (B)). In contrast, derivatives (XIV (A)) and (XIV (C)) exhibited low cytotoxicity against all cancer cell lines, with ID_50_ values ranging from 32–105 µM. Among the 1,3,4-oxadiazole derivatives, compounds (XV (E)) and (XV (F)) with *p*-bromophenyl or pyridyl substituents in the C-5 position had the strongest cytotoxicity against MT-4 (ID_50_ = 11 µM and ID_50_ =16 µM, respectively) and U-937 human cancer cells (ID_50_ = 17µM and ID_50_ =15 µM, respectively). The cytotoxic activity against CEM-13 cell line was moderate for both oxadiazole derivatives (ID_50_ = 33 µM for derivative (XV (E)) and ID_50_ = 37 µM derivative (XV (F)), respectively. Regarding the inhibitory activities of compounds (XV (A–F)), ID_50_ values were much higher than those of betulonic acid, suggesting that these derivatives had low cytotoxicity (ID_50_ ranged from 28 to 120 µM) as shown in Table 2.

The cytotoxicity of these compounds was significantly influenced by the nature of the substitutes at oxadiazole C-5 [117].

The cytotoxicity of betulonic acid conjugated with triphenylphosphonium salts (TPP) against human breast cancer (MCF-7), human prostate adenocarcinoma (PC-3), and normal human skin fibroblasts (HSF) was studied by Tsepaeva et al. Synthesis of betulonic acid-derived TPP salts was conducted by the reaction of halo alkyl esters with triphenylphosphine. According to MTT assay, after 72 h incubation, 9-triphenylphosphoniononyl 3-oxolup-20(29)-en-28-oate bromide (XVI) exhibited the highest inhibitory activity against MCF-7 cells (IC_50_ ≈ 0.3 µM), comparable to doxorubicin (IC_50_ ≈ 0.4 µM). In terms of anticancer activity against PC-3 cells, the 2-triphenylphosphonioethoxyethyl 3-oxolup-20(29)-en-28-oate bromide derivative (XVII) exerted the strongest cytotoxicity (IC_50_ ≈ 0.4 µM). This study has shown that these derivatives have improved the antiproliferative activity on cancer cells and not on normal HSF cells. Such selectivity may be due to the enhanced uptake of triterpenoids by cancer cells, presumably attributable to the TPP moiety [118].

The pro-apoptotic and cytotoxic activity of betulonic acid derivatives was reported by Csuk et al. The research groups have synthesized C(2)-methylene derivatives either by Mannich reactions, accompanied by *β*-elimination, or by aldol condensation reactions. Cytotoxicity was determined by sulforhodamine B assay for various human cancer cell lines: melanoma (518A2), cervical cancer (A431), head and neck tumor (A253, FADU), lung carcinoma (A549), ovarian cancer (A2780), colon cancer (DLD-1, HCT-8, HCT-116, HT-29, SW480), anaplastic thyroid cancer (8505c, SW1736), mammary carcinoma (MCF-7), liposarcoma (LIPO), and mouse fibroblasts (NiH3T3). According to the reported results, 2-methylene-3-oxolup-20(29)en-28-oic acid (XVIII) presented excellent cytotoxicity against all cancer cell lines, with IC_50_ values ranging from 0.2–0.6 μM after 96 h of exposure. The 2-methylene-28-oxo-28-thiomorpholin-4-yl-lup-20(29)en-3-one (XIX) compound also displayed promising results as an anticancer agent according to the IC_50_ values within the range of 1–2 μM. Moreover, in order to increase the bioavailability of the methyl 2-methylene-3-oxolup-20(29)en-28-oate (XX), the authors encapsulated the compound into soybean lecithin liposomes (XX (E)), thus leading to a threefold rise in cytotoxicity. 2-methylene-3-oxolup-20(29)en-28-oic acid (XVIII), methyl 2-methylene-3-oxolup-20(29)en-28-oate(XX) and methyl (2*α*)-2-{[(2-hydroxyethyl)thio]methyl}-3-oxolup- 20(29)en-28-oate (XXI) were also examined in terms of pro-apoptotic activity; they induced DNA fragmentation in colon cancer cell line SW480, thereby providing strong signs of apoptosis. In addition, acridine orange/ethidium bromide staining of A549 lung carcinoma cells treated with these derivatives also showed induced apoptosis [119].

Kazakova et al. have reported the biological activity of C-28-imidazolides of betulonic acid, containing 3-oxo-, 3-hydroxyimino-, and 2-cyano-2,3-seco-4(23)-ene fragments in cycle A. Firstly, they assessed the cytotoxicity of the imidazolides (cycle A) using different human tumor cell lines (lungs, colon, central nervous system, ovary, renal, prostate, mammary gland, melanoma, leukemia), dyed with sulforhodamine B, and treated with 10 μM of the derivatives for 48 h. The results have shown that the imidazolide of 3-hydroxyimino-lup-20(29)-en-28-carboxylic acid (cycle A) (XXII) was the most efficient in inhibiting the cell growth of all six lung cancer cell lines, all seven large intestine cancer cell lines, all six leukemia cell lines, all six melanoma cell lines, all four breast cancer cell lines, two nervous system cancer cell lines, one prostate cancer cell line, three ovarian cancer cell lines, and five renal cancer cell lines. The cell survival rates are depicted in Table 2. Moreover, cell death was found in three cell lines: lung cancer cell line (NCI-H460), colon cancer cell line (COLO 205), and leukemia cell line (HL-60[TB]) [107]. An overview of the anticancer activity of betulonic acid derivatives is presented in Figure 2.

**Figure 3:**
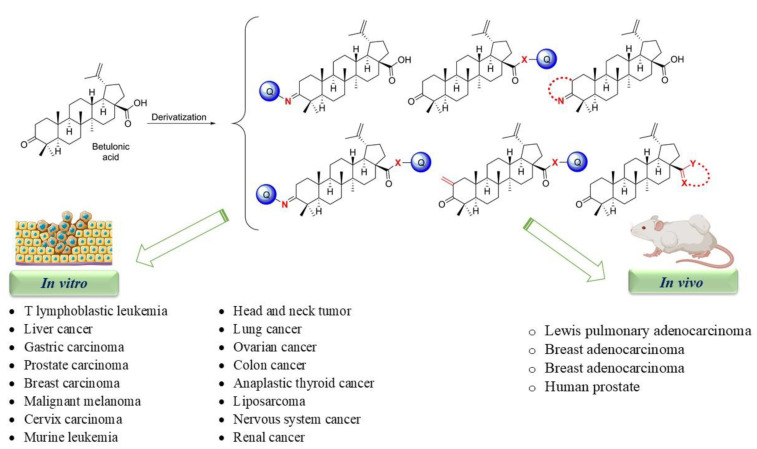
A snap shot of the in vitro and in vivo anticancer effects of the most active derivatives of betulonic acid.

Owing to the hydrophobic properties of betulonic acid, its in vivo anti-cancer effectiveness has not been intensively studied (Figure 3). In a research performed by Saxena et al., they investigated the in vivo activity of hydrophilic lysinated betulonic acid on LNcaP prostate cancer cells xenografts in athymic mice (Table 3). In contrast to the control group, the lysinated betulonic acid injected mice displayed a 92% inhibition of tumor development. Moreover, histological analyses of the tumors revealed the lack of dividing cells, demonstrating the anticancer activity of the lysinated-betulonic acid [116].

Based on the remarkable results obtained for the imidazolide of 3-hydroxyimino-lup-20(29)-en-28-carboxylic acid (XXII), Kazakova et al. have studied its antineoplastic activity in vivo on two ascites tumors (Ehrlich tumor and P388 lympholeukemia) and three solid tumors (breast adenocarcinoma [Ca755], colon adenocarcinoma [AKATOL], and Lewis lung cancer [LLC]) inoculated in BDF1 hybrids (DBA2 × C57B1/6J), BALB/C, and DBA mice. This chemical derivative injected intraperitoneally, five times daily at a dosage of 70 mg/kg, had no antineoplastic effect neither on the Ehrlich ascites tumor nor P388 lympholeukemia (at a dose of 30 and 50 mg/kg), respectively. Interestingly, the administration of 50 mg/kg through the same procedure suppressed the development of breast adenocarcinoma (Ca755) by 56–77%. The result was maintained without an improvement in life duration until day eight of the study. In mice with large intestinal adenocarcinoma treated by the same procedure, a 53% decrease in tumor growth was seen only after 5 days of therapy without any change in the mice’s lifespan [107].

The research study conducted by Zhukova et al. examined the effects of betulonic acid and its methyl esters derivatives on the pathological modifications in the kidneys of the C57BL/6 mice, presenting Lewis pulmonary adenocarcinoma. Compounds were administered intraperitoneally in a dosage of 50 mg/kg for 8 days. After 8 days, mice were decapitated under ethereal anesthesia and sections of the treated kidneys were analyzed using periodic acid–Schiff reaction-hematoxylin and eosin-orange G dyeing. These chemical derivatives had a beneficial impact on the course of paraneoplastic nephropathy; they decreased the volume density of epithelial cell necrosis at 56%. On average, the number of cells with edematic cytoplasm, vesicular lipid infiltrate, and disrupted brush lymbus fell by 8–13% in comparison to untreated tumoral cells [120].

## 4. Conclusions

Data presented in this work show that following structural modifications, which involved the introduction of amide, thiol, and piperidine groups, amplification of the in vitro anticancer potential of betulonic acid can be achieved. Studies published in the state of the art literature revealed that betulonic acid derivatives possess an important in vitro cytotoxic and pro-apoptotic activity. However, the number of in vivo studies is limited. The screening of scientific literature on this topic indicates a demand for in-depth in vivo data regarding the effect and mechanism of action of the betulonic acid derivatives in order to benefit from these structures in different therapeutic protocols designated for the management of various types of cancer.

## Figures and Tables

**Table 1:** Betulonic acid derivatives.

Number	Derivative	Substituent	Reference
I	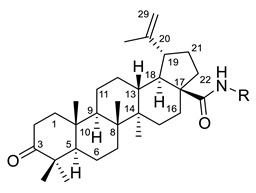	R = (CH_2_)_7_COOH (A) R = (CH_2_)_8_COOH (B) R = (CH_2_)_10_COOH (C) R = (CH_2_)_8_CONHCH(Ph)CH_2_COOH (D)	[112]
II	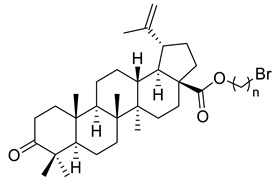	*n* = 2 (A) *n* = 3 (B)	[113]
III	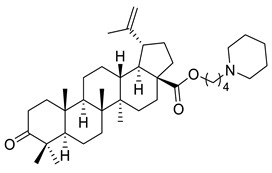		[113]
IV	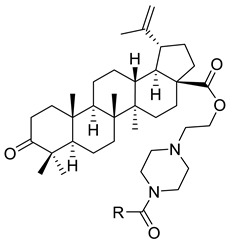	R = C_6_H_5_ (A) R = 3-F-C_6_H_4_ (B)	[113]
V	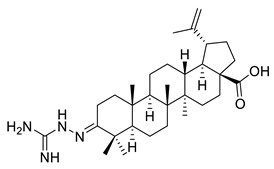		[114]
VI	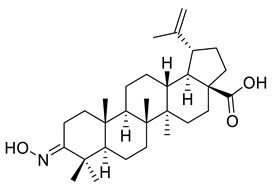		[114]
VII	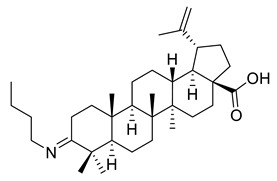		[114]
VIII	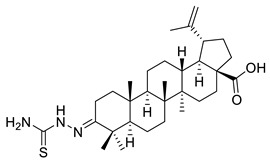		[114]
IX	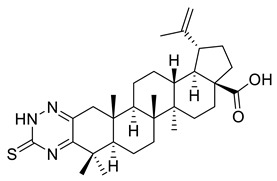		[3]
X	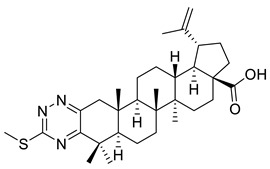		[3]
XI	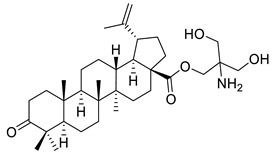		[3]
XII	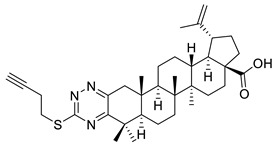		[115]
XIII	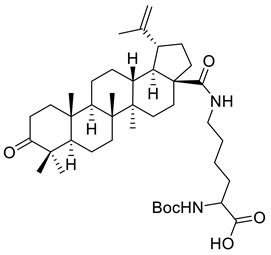		[116]
XIV	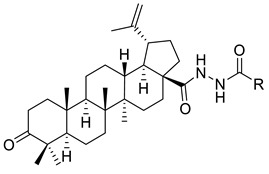	R = COOEt (A) R = C_6_H_5_ (B) R = 3,4-OMe-C_6_H_3_ (C) R = 2-F-3-Cl-6-CF_3_-C_6_H_2_ (D) R =4-Br-C_6_H_4_ (E) R = Py-4-yl (F)	[117]
XV	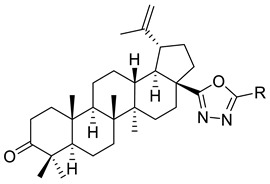	R = COOEt (A) R = C_6_H_5_ (B) R = 3,4-OMe-C_6_H_3_ (C) R = 2-F-3-Cl-6-CF_3_-C_6_H_2_ (D) R =4-Br-C_6_H_4_ (E) R = Py-4-yl (F)	[117]
XVI	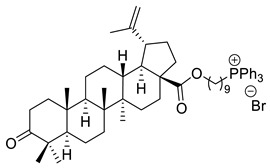		[118]
XVII	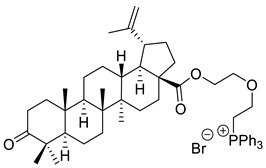		[118]
XVIII	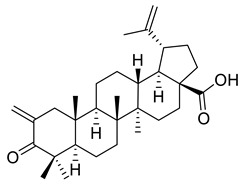		[119]
XIX	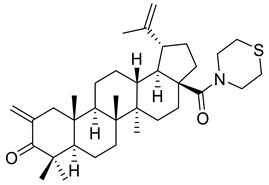		[119]
XX	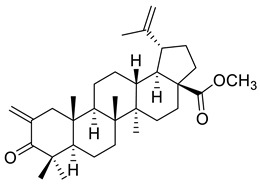		[119]
XXI	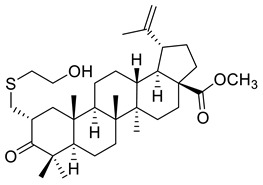		[119]
XXII	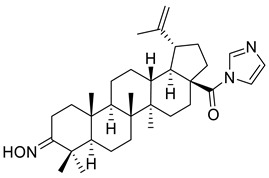		[107]

**Table 2:** Role of betulonic acid derivatives among various cancer cell lines.

Compound	Type Cancer Cell Line	Cell Line	In Vitro	Conclusion	Reference
A	T-cell leukemia	MT-4	Compound concentration inhibiting by 50% the tumor-cell viability (ID_50_) ≈ 25 µg/mL	Betulonic acid amides inhibited growth of tumor cells and induced apoptosis Betulonic acid amides inhibited growth of tumor cells and induced apoptosis	[112]
		MOLT-4	ID_50_ ≈ 10 µg/mL		
		CEM	ID_50_ ≈ 9 µg/mL		
	Liver cancer	Hep G2	ID_50_ ≈ 32 µg/mL		[112]
I B	T-cell leukemia	MT-4	ID_50_ ≈ 20 µg/mL		
		MOLT-4	ID_50_ ≈ 16 µg/mL		
		CEM	ID_50_ ≈ 13 µg/mL		
	Liver cancer	Hep G2	ID_50_ ≈ 32 µg/mL		
I C	T-cell leukemia	MT-4	ID_50_ ≈ 32 µg/mL		
		MOLT-4	ID_50_ ≈ 10 µg/mL		
		CEM	ID_50_ ≈ 8 µg/mL		
	Liver cancer	Hep G2	ID_50_ ≈ 32 µg/mL		
I D	T-cell leukemia	MT-4	ID_50_ ≈ 4 µg/mL		
		MOLT-4	ID_50_ ≈ 5 µg/mL		
		CEM	ID_50_ ≈ 7 µg/mL		
	Liver cancer	Hep G2	ID_50_ ≈ 25 µg/mL		
II A	Gastric carcinoma	MGC-803	Half maximal inhibitory concentration (IC_50_) = above 20 µM Half maximal inhibitory concentration (IC_50_) = above 20 µM	Antiproliferative activity was significantly reduced Antiproliferative activity was significantly reduced	[113]
	Prostate carcinoma	PC3			
	Breast carcinoma	Bcap-37			
		MCF-7			
	Malignant melanoma	A375			
II B	Gastric carcinoma	MGC-803			
	Prostate carcinoma	PC3			
	Breast carcinoma	Bcap-37			[113]
		MCF-7			
	Malignant melanoma	A375			
III	Gastric carcinoma	MGC-803	IC_50_ ≈ 4 µM	Derivative inhibited cell proliferation and induced cell apoptosis	[113]
	Prostate carcinoma	PC3	IC_50_ ≈ 6 µM		
	Breast carcinoma	Bcap-37	IC_50_ ≈ 4 µM		
		MCF-7	IC_50_ ≈ 5 µM		
	Malignant melanoma	A375	IC_50_ ≈ 8 µM		
IV A	Gastric carcinoma	MGC-803	IC_50_ ≈ 5 µM	Compounds exhibited antiproliferative ability	[113]
	Prostate carcinoma	PC3	IC_50_ ≈ 4 µM		
	Breast carcinoma	Bcap-37	IC_50_ ≈ 7 µM		
		MCF-7	IC_50_ ≈ 5 µM		
	Malignant melanoma	A375	IC_50_ ≈ 6 µM		
IV B	Gastric carcinoma	MGC-803	IC_50_ ≈ 5 µM		
	Prostate carcinoma	PC3	IC_50_ ≈ 7 µM		
	Breast carcinoma	Bcap-37	IC_50_ ≈ 6 µM		
		MCF-7	IC_50_ ≈ 5 µM		
	Malignant melanoma	A375	IC_50_ ≈ 8 µM		
V	Cervix adenocarcinoma	HeLa	Inhibition percentage (Inhib) = 60%	Compound showed weak cytotoxic activity	[114]
	Skin carcinoma	A431	Inhib ≈ 20%		
	Ovarian carcinoma	A2780	Inhib ≈ 46%		
	Breast adenocarcinoma	MCF-7	Inhib = 40%		
VI	Cervix adenocarcinoma	HeLa	Inhib ≈ 26%	Compounds revealed a cytotoxic effect on HeLa, A431, A2780, MCF-T tumor cell lines, except for derivatives VI and VIII. They didn’t have cytotoxic activity on the A431 cell line.	[114]
	Skin carcinoma	A431	Inhib ≈ −21%		
	Ovarian carcinoma	A2780	Inhib ≈ 51%		
	Breast adenocarcinoma	MCF-7	Inhib ≈ 29%		
VII	Cervix adenocarcinoma	HeLa	Inhib ≈ 55%		
	Skin carcinoma	A431	Inhib ≈ 20%		
	Ovarian carcinoma	A2780	Inhib ≈ 41%		
	Breast adenocarcinoma	MCF-7	Inhib = 40%		
VIII	Cervix adenocarcinoma	HeLa	Inhib ≈ 71%		
	Skin carcinoma	A431	Inhib ≈ −6%		
	Ovarian carcinoma	A2780	Inhib ≈ 75%		
	Breast adenocarcinoma	MCF-7	Inhib ≈ 71%		
IX	Murine leukemia	L1210	IC_50_ = 196 µM	Derivative did not inhibit tumor cell proliferation.	[3]
	T-cell leukemia	CEM	IC_50_ = 204 µM		
	Cervix carcinoma	HeLa	IC_50_ = 160 µM		
X	Murine leukemia	L1210	IC_50_ ≈ 7 µM	Compounds exhibited antiproliferative activity	[3]
	T-cell leukemia	CEM	IC_50_ ≈ 3 µM		
	Cervix carcinoma	HeLa	IC_50_ ≈ 7 µM		
XI	Murine leukemia	L1210	IC_50_ ≈ 5 µM		
	T-cell leukemia	CEM	IC_50_ ≈ 1 µM		
	Cervix carcinoma	HeLa	IC_50_ ≈ 9 µM		
XII	Melanoma	518A2	IC_50_ ≈ 11 µM	Compound presented cytotoxic activity on all tumor cell lines and induced apoptosis on colon cancer cell line	
	Head and neck tumor	A253	IC_50_ ≈ 11 µM		[115]
	Cervical cancer	A431	IC_50_ ≈ 10 µM		
	Lung cancer	A549	IC_50_ ≈ 11 µM		
	Ovarian cancer	A2780	IC_50_ ≈ 10 µM		
	Colon cancer	HT-29	IC_50_ ≈ 9 µM		
	Breast carcinoma	MCF-7	IC_50_ ≈ 11 µM		
	Anaplastic thyroid tumor	SW1736	IC_50_ ≈ 10 µM		
XIII	Prostate cancer	LNCaP	Inhib ≈ 96%	Boc-lysinated-betulonic acid inhibited the growth of prostate cancer cells.	[116]
		DU-145	Inhib ≈ 93%		
		PC3	Inhib = 77%		
XIV A	Human tumor cells	CEM-13	ID_50_ = 105 µM	Betulonic acid hydrazides presented low cytotoxicity	[117]
	T-cell leukemia	MT-4	ID_50_ = 80 µM		
	Human monocytes	U-937	ID_50_ = 33 µM		
XIV B	Human tumor cells	CEM-13	ID_50_ = 72 µM		
	T-cell leukemia	MT-4	ID_50_ = 32 µM		
	Human monocytes	U-937	ID_50_ = 22 µM		
XIV C	Human tumor cells	CEM-13	ID_50_ = 41 µM		
	T-cell leukemia	MT-4	ID_50_ = 44 µM		
	Human monocytes	U-937	ID_50_ = 32 µM		
XIV D	Human tumor cells	CEM-13	ID_50_ = 32 µM	Betulonic acid hydrazides presented the highest cytotoxic activity in several cancer cell lines	[117]
	T-cell leukemia	MT-4	ID_50_ = 9 µM		
	Human monocytes	U-937	ID_50_ = 12 µM		
XIV E	Human tumor cells	CEM-13	ID_50_ = 35 µM		
	T-cell leukemia	MT-4	ID_50_ = 14 µM		
	Human monocytes	U-937	ID_50_ = 23 µM		
XIV F	Human tumor cells	CEM-13	ID_50_ = 57 µM	Betulonic acid hydrazide presented moderate cytotoxic activity in several cancer cell line	[117]
	T-cell leukemia	MT-4	ID_50_ = 19 µM		
	Human monocytes	U-937	ID_50_ = 24 µM		
XV A	Human tumor cells	CEM-13	ID_50_ = 120 µM	The 1,3,4-oxadiazole derivatives with ethoxycarbonyl, phenyl and methoxyphenyl substituents exhibited low cytotoxic activity in several cancer cell line	[117]
	T-cell leukemia	MT-4	ID_50_ = 79 µM		
	Human monocytes	U-937	ID_50_ = 26 µM		
XV B	Human tumor cells	CEM-13	ID_50_ = 63 µM		
	T-cell leukemia	MT-4	ID_50_ = 62 µM		
	Human monocytes	U-937	ID_50_ = 35 µM		
XV C	Human tumor cells	CEM-13	ID_50_ = 52 µM		
	T-cell leukemia	MT-4	ID_50_ = 45 µM		
	Human monocytes	U-937	ID_50_ = 28 µM		
XV E	Human tumor cells	CEM-13	ID_50_ = 33 µM	The 1,3,4-oxadiazole derivatives with *p*-bromophenyl or pyridyl substituents exhibited the highest cytotoxic activity	[117]
	T-cell leukemia	MT-4	ID_50_ ≈ 11 µM		
	Human monocytes	U-937	ID_50_ ≈ 19 µM		
XV F	Human tumor cells	CEM-13	ID_50_ = 37 µM		
	T-cell leukemia	MT-4	ID_50_ ≈ 16 µM		
	Human tumor cells	CEM-13	ID_50_ ≈ 15 µM		
XVI	Breast carcinoma	MCF-7	IC_50_ = 0.3 µM	Betulonic acid C-28-triphenylphosphonium derivatives presented great cytotoxic activity in several cancer cell lines	[118]
	Prostate cancer	PC-3	IC_50_ ≈ 0.9 µM		
XVII	Breast carcinoma	MCF-7	IC_50_ ≈ 0.9 µM		
	Prostate cancer	PC-3	IC_50_ ≈ 0.4 µM		
XVIII	Melanoma	518A2	IC_50_ = 0.5 µM	C (2)-methylene derivative presented cytotoxic activity on all tumor cell lines and also pro-apoptotic activity on SW480 and A549 cell lines	
	Cervical cancer	A431	IC_50_ = 0.2 µM		
	Head and neck tumor	A253	IC_50_ = 0.4 µM		
		FADU	IC_50_ = 0.5 µM		
	Lung carcinoma	A549	IC_50_ = 0.6 µM		[119]
	Ovarian cancer	A2780	IC_50_ = 0.6 µM		
	Colon cancer	DLD-1	IC_50_ = 0.4 µM		
		HCT-8	IC_50_ = 0.2 µM		
		HCT-116	IC_50_ = 0.2 µM		
		HT-29	IC_50_ = 0.4 µM		
		SW480	IC_50_ = 0.4 µM		
	Anaplastic thyroid cancer	8505c	IC_50_ = 0.6 µM		
		SW1736	IC_50_ = 0.4 µM		
	Breast carcinoma	MCF-7	IC_50_ = 0.3 µM		
	Liposarcoma	LIPO	IC_50_ = 0.6 µM		
	Mouse fibroblasts	NiH3T3	IC_50_ = 0.8 µM		
XIX	Melanoma	518A2	IC_50_ ≈ 2 µM	C(2)-methylene derivative presented cytotoxic activity on all tumor cell lines	[119]
	Cervical cancer	A431	IC_50_ ≈ 1 µM		
	Head and neck tumor	A253	IC_50_ ≈ 1 µM		
		FADU	IC_50_ ≈ 2 µM		
	Lung carcinoma	A549	IC_50_ ≈ 2 µM		
	Ovarian cancer	A2780	IC_50_ ≈ 1 µM		
	Colon cancer	DLD-1	IC_50_ ≈ 2 µM		
		HCT-8	IC_50_ ≈ 2 µM		
		HCT-116	IC_50_ ≈ 1 µM		
		HT-29	IC_50_ ≈ 2 µM		
		SW480	IC_50_ ≈ 2 µM		
	Anaplastic thyroid cancer	8505c	IC_50_ ≈ 2 µM		
		SW1736	IC_50_ ≈ 2µM		
	Breast carcinoma	MCF-7	IC_50_ ≈ 1 µM		
	Liposarcoma	LIPO	IC_50_ ≈ 2 µM		
	Mouse fibroblasts	NiH3T3	IC_50_ ≈ 2 µM		
XX	Melanoma	518A2	IC_50_ ≈ 4 µM	C (2)-methylene derivative presented a low cytotoxic activity on all tumor cell lines and also pro-apoptotic activity on SW480 and A549 cell lines	[119]
	Cervical cancer	A431	IC_50_ ≈ 5 µM		
	Head and neck tumor	A253	IC_50_ ≈ 4 µM		
		FADU	IC_50_ ≈ 8 µM		
	Lung carcinoma	A549	IC_50_ ≈ 5 µM		
	Ovarian cancer	A2780	IC_50_ ≈ 5 µM		
	Colon cancer	DLD-1	IC_50_ ≈ 6 µM		
		HCT-8	IC_50_ = 9 µM		
		HCT-116	IC_50_ ≈ 5 µM		
		HT-29	IC_50_ ≈ 6 µM		
		SW480	IC_50_ ≈ 5 µM		
	Anaplastic thyroid cancer	8505c	IC_50_ ≈ 5 µM		
		SW1736	IC_50_ ≈ 5 µM		
	Breast carcinoma	MCF-7	IC_50_ ≈ 4 µM		
	Liposarcoma	LIPO	IC_50_ ≈ 5 µM		
	Mouse fibroblasts	NiH3T3	IC_50_ ≈ 5 µM		
XX E	Melanoma	518A2	IC_50_ ≈ 2 µM	C (2)-methylene derivative encapsulated in liposomes had a threefold rise in cytotoxic activity on all tumor cell lines compared to the non-encapsulated one	
	Cervical cancer	A431	IC_50_ ≈ 2 µM		
	Head and neck tumor	A253	IC_50_ ≈ 2 µM		
		FADU	IC_50_ ≈ 3 µM		
	Lung carcinoma	A549	IC_50_ ≈ 2 µM		
	Ovarian cancer	A2780	IC_50_ ≈ 3 µM		
	Colon cancer	DLD-1	IC_50_ ≈ 3 µM		
		HCT-8	IC_50_ ≈ 2 µM		
		HCT-116	IC_50_ ≈ 2 µM		[119]
		HT-29	IC_50_ ≈ 2 µM		
		SW480	IC_50_ ≈ 2 µM		
	Anaplastic thyroid cancer	8505c	IC_50_ ≈ 2 µM		
		SW1736	IC_50_ ≈ 2 µM		
	Breast carcinoma	MCF-7	IC_50_ ≈ 2 µM		
	Liposarcoma	LIPO	IC_50_ ≈ 2 µM		
	Mouse fibroblasts	NiH3T3	IC_50_ ≈ 2 µM		
XXII	Lung cancer	A549/ATCC	Survival rate = 0.05%	The 3-hydroxyimino derivative of betulonic acid inhibited the cell growth and caused death of several cancer cell linesThe 3-hydroxyimino derivative of betulonic acid inhibited the cell growth and caused death of several cancer cell lines	[107]
		HOP-62	Survival rate ≈ 26%		
		NCI-H23	Survival rate ≈ 17%		
		NCI-H322M	Survival rate ≈ 20%		
		NCI-H460	Survival rate ≈ −2%		
		NCI-H522	Survival rate ≈ 7%		
	Colon cancer	COLO 205	Survival rate ≈ −58%		
		HCC-2998	Survival rate ≈ 7%		
		HCT-116	Survival rate ≈ 5%		
		HCT-15	Survival rate ≈ 16%		
		HT29	Survival rate ≈ 13%		
		KM12	Survival rate ≈ 21%		[107]
		SW-620	Survival rate ≈ 23%		
	Leukemia	CCRF-CEM	Survival rate = 11%		
		HL-60(TB)	Survival rate ≈ −15%		
		K-562	Survival rate ≈ 5%		
		MOLT-4	Survival rate ≈ 6%		
		RPMI-8226	Survival rate ≈ 9%		
		SR	Survival rate ≈ 6%		
	Melanoma	LOX IMVI	Survival rate ≈ 3%		
		MDA-MB-435	Survival rate ≈ 28%		
		SK-MEL-28	Survival rate ≈ 28%		
		SK-MEL-5	Survival rate ≈ 15%		
		UACC-257	Survival rate ≈ 12%		
		UACC-62	Survival rate ≈ 26%		
	Breast cancer	MCF7	Survival rate ≈ 2%		
		MDA-MB-231/ATCC	Survival rate ≈ 14%		[107]
		T-47D	Survival rate ≈ 25%		
		MDA-MB-468	Survival rate ≈ 8%		
	Nervous system cancer	SF-539	Survival rate ≈ 22%		
		U251	Survival rate ≈ 15%		
	Prostate cancer	PC-3	Survival rate ≈ 26%		
	Ovarian cancer	OVCAR-3	Survival rate ≈ 25%		
		OVCAR-8	Survival rate ≈ 17%		
		NCI/ADR-RES	Survival rate ≈ 14%		
	Renal cancer	786-0	Survival rate ≈ 32%		
		CAKI-1	Survival rate ≈ 13%		
		SN12C	Survival rate ≈ 19%		
		TK-10	Survival rate ≈ 18%		
		UO-31	Survival rate ≈ 10%		

**Table 3:** In vivo studies of betulonic acid derivatives.

Compound	Experimental Animal Model	Injected Tumor Cells	Concentration	Conclusion	Reference
XIII	Athymic male mice	Human prostate LNCaP cells	30 mg/kg	Boc-lysinated-betulonic acid inhibited the growth of LNCaP xenograft tumors.	[116]
XXII	BDF1 hybrids (DBA2 × C57B1/6J), BALB/C, and DBA	Ehrlich tumor	70 mg/kg	Imidazolide of betulonic acid had no antineoplastic effect on	[107]
		Lympholeukemia P388	30 mg/kg and 50 mg/kg		
		Breast adenocarcinoma Ca755	50 mg/kg	Imidazolide of betulonic acid inhibited the tumoral growth	
		Colon adenocarcinoma AKATOL	50 mg/kg		
		Lewis lung cancer LLC	50 mg/kg	Imidazolide of betulonic acid had no curative effect	
XXIII	C57BL/6 mice	Lewis pulmonary adenocarcinoma	50 mg/kg	Methyl ester derivatives of betulonic acid decreased the volume density of epithelial cell necrosis at 56%	[120]

## Data Availability

No new data were created or analyzed in this study.

## Abbreviations

ACh	Acetylcholine
DMSO	Dimethyl sulfoxide
DNA	Deoxyribonucleic acid
EMA	European Medicines Agency
FDA	Food and Drug Administration
HeLa	Cervical adenocarcinoma
IC_50_	Half maximal inhibitory concentration
ID_50_	Compound concentration inhibiting by 50% the tumor-cell viability
IL-1*β*	Interleukin 1 beta
IL-6	Interleukin 6
MDA-MB-231	Triple-negative breast cancer
miRNA-27	Microrna
mRNA	Messenger RNA
MTT	3-(4,5-dimethylthiazol-2-yl)-2,5-diphenyl tetrazolium bromide
PBMC	Peripheral mononuclear blood cells
PBS	Phosphate-buffered saline
PT	Pentacyclic triterpenoids
ROS	Reactive oxygen species
SP	Specificity protein
SREBP	Sterol regulatory element-binding proteins
TNF-α	Tumor Necrosis Factor-α
TPP	Triphenylphosphonium
VEGFR	Vascular endothelial growth factor
